# Comparison of delirium detection tools in acute care: A rapid review

**DOI:** 10.1007/s00391-021-02003-5

**Published:** 2022-01-14

**Authors:** Simone Brefka, Gerhard Wilhelm Eschweiler, Dhayana Dallmeier, Michael Denkinger, Christoph Leinert

**Affiliations:** 1 Institute for Geriatric Research, Ulm University, Ulm, Germany; 2 Geriatric Center Ulm/Alb Donau, Ulm, Germany; 3 Agaplesion Bethesda Hospital Ulm, Zollernring 26, 89073 Ulm, Germany; 4 Geriatric Center, University Hospital Tuebingen, Tuebingen, Germany; 5 University Hospital for Psychiatry and Psychotherapy Tuebingen, Tuebingen, Germany; 6 Dept. of Epidemiology, Boston University School of Public Health, Boston, USA

**Keywords:** Practicability, Assessment, Screening, Confusion assessment method, 4As test, Praktikabilität, Assessment, Screening, Confusion Assessment Method, 4As-Test

## Abstract

**Background:**

Delirium is a frequent psychopathological syndrome in geriatric patients. It is sometimes the only symptom of acute illness and bears a high risk for complications. Therefore, feasible assessments are needed for delirium detection.

**Objective and methods:**

Rapid review of available delirium assessments based on a current Medline search and cross-reference check with a special focus on those implemented in acute care hospital settings.

**Results:**

A total of 75 delirium detection tools were identified. Many focused on inattention as well as acute onset and/or fluctuating course of cognitive changes as key features for delirium. A range of assessments are based on the confusion assessment method (CAM) that has been adapted for various clinical settings. The need for a collateral history, time resources and staff training are major challenges in delirium assessment. Latest tests address these through a two-step approach, such as the ultrabrief (UB) CAM or by optional assessment of temporal aspects of cognitive changes (4 As test, 4AT). Most delirium screening assessments are validated for patient interviews, some are suitable for monitoring delirium symptoms over time or diagnosing delirium based on collateral history only.

**Conclusion:**

Besides the CAM the 4AT has become well-established in acute care because of its good psychometric properties and practicability. There are several other instruments extending and improving the possibilities of delirium detection in different clinical settings.

**Supplementary Information:**

The online version of this article (10.1007/s00391-021-02003-5) contains supplementary material, which is available to authorized users.

## Abstract (de)

**Hintergrund:**

Das Delir ist ein häufiges psychopathologisches Syndrom bei geriatrischen Patient:innen. Teilweise stellt es das einzige Symptom einer Akuterkrankung dar und geht mit einem hohen Komplikationsrisiko einher. Zur Erfassung sind daher praktikable Assessments wichtig.

**Zielsetzung und Methodik:**

Rapid Review basierend auf einer aktuellen Medline-Suche und Querverweisanalyse zu verfügbaren Delir-Assessments mit Fokus auf den Einsatz im akutmedizinischen Bereich.

**Ergebnisse:**

Insgesamt 75 Delir-Assessments wurden identifiziert. Viele detektierten als Schwerpunkt Aufmerksamkeitsstörungen sowie den für ein Delir charakteristischen akuten Beginn/fluktuierenden Verlauf. Eine Reihe von Assessments basieren auf der Confusion Assessment Method (CAM), die für verschiedene klinische Bereiche modifiziert wurde. Die Notwendigkeit zur Einholung einer Fremdanamnese, limitierte Zeitressourcen sowie die Schulung von Assessoren stellen bei vielen Assessments eine Herausforderung dar. Dieser wird u. a. dadurch begegnet, dass ein kurzes Screening einem ausführlicheren Assessment vorgeschaltet wird (z. B. ultra-brief CAM, UB-CAM). Alternativ muss bei manchen Assessments die Fremdanamnese nur fakultativ erhoben werden (z. B. 4 As Test, 4AT). Die meisten sind für das Screening von Patient:innen geeignet, es gibt aber auch Assessments, die eine Schweregradeinteilung oder ein Monitoring von Delir-Symptomen ermöglichen oder auch nur auf einer Fremdanamnese basieren.

**Diskussion:**

Neben der CAM hat sich der 4AT in der Akutmedizin aufgrund der guten Praktikabilität und psychometrischen Charakteristika etabliert. Daneben existieren weitere geeignete Assessments, die einen zielgerichteten Einsatz in verschiedenen klinischen Bereichen erlauben und die Möglichkeiten des Delir-Assessments erweitern.

## Introduction

Delirium is a frequent psychopathological and cognitive syndrome in geriatric patients and is associated with a high risk of acute care complications and negative outcomes [37]. It is the most important presenting phenotype of acute encephalopathy [61]. Therefore, delirium detection is an important part of evaluation of geriatric patients and feasible assessments are needed.

## Background

Multiple risk factors contribute to the development of delirium and a multifactorial origin is common [28]. Various prevention and treatment options for delirium exist and range from optimizing risk factors (e.g. reducing anticholinergic medication) to the treatment of precipitating factors (e.g. pneumonia, electrolyte imbalances, pain) and the implementation of nonpharmacological multimodal care models [37]. For the adequate administration of preventive measures and treatment early identification of patients at high risk for and with delirium is essential; however, presentation of delirium can be heterogeneous and identification challenging.

### Psychopathology and cognitive alterations in delirium

For a tailored use of delirium screening instruments, it is important to understand the different aspects of delirium as a psychopathological syndrome and its diagnostic criteria. In the International Classification of Diseases 10th edition (ICD-10) the syndrome of delirium is described as a rather variable combination of different psychopathological disturbances with a non-specific organic etiology (Table 1; [53]). Besides altered consciousness, an alteration of attention, perception, (logical) thinking, memory, psychomotor behavior, emotion or the sleep-wake schedule might be present.

**Table 1:** Comparison of ICD-10 and ICD-11 for delirium diagnosis

ICD-10—F05 Delirium not induced by alcohol and other psychoactive substances	ICD-11—6D70 Neurocognitive disorders: delirium
An etiologically nonspecific organic cerebral syndrome characterized by concurrent disturbances of consciousness and at least two of the following domains: attention, perception, thinking, memory, psychomotor behavior, emotion, or sleep-wake schedule. The duration is variable and the degree of severity ranges from mild to very severe	Delirium is characterized by: 1. disturbed attention (i.e., reduced ability to direct, focus, sustain, and shift attention) and 2. awareness (i.e., reduced orientation to the environment) that develops over a short period of time and tends to fluctuate during the course of a day, accompanied by other cognitive impairment such as: memory deficit, disorientation, or impairment in language, visuospatial ability, or perception. Disturbance of the sleep-wake cycle (reduced arousal of acute onset or total sleep loss with reversal of the sleep-wake cycle) may also be present. The symptoms are attributable to a disorder or disease not classified under mental and behavioral disorders or to substance intoxication or withdrawal or to a medication

Different subtypes of delirium exist: hyperactive delirium accounts for 20% of cases and is characterized by psychomotor agitation and restlessness [43]. It often includes pulling out tubes and is disrupting for hospital procedures, and is therefore an eye-catcher diagnosis for hospital professionals. In contrast hypoactive delirium, accounting for 30% of cases, presents with reduced psychomotor activity and patients are often misdiagnosed with depression or severe dementia. About 45% of delirious patients can show both hyperactive and hypoactive symptoms indicating a fluctuating course of disease.

Delirium can also be accompanied by psychotic symptoms. More than 40% of patients display predominantly optical hallucinations or delusions, such as being poisoned or persecuted [45].

The reference standard for delirium diagnosis has most commonly been the psychopathological examination through a skilled psychiatrist. The delirium rating scale in its original and revised version (DRS-R-98) sticks very closely to the various possibilities of psychopathological disturbances that can be found in delirium and tries to operationalize this psychopathological examination [58, 59]. Therefore, it is time-consuming and relatively complex to administer.

### Development of the confusion assessment method (CAM)

**Fig. 1:**
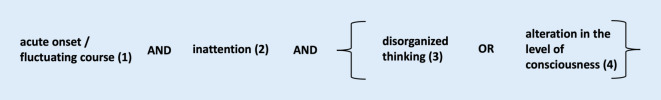
Confusion assessment method (CAM) algorithm. Criteria 1 and 2 as well as either criterion 3 or criterion 4 must be present for a CAM-based diagnosis of delirium

In 1990 the first version of the confusion assessment method (CAM) was introduced, which is based on four core criteria [2, 29]. These criteria were derived from the Diagnostic and Statistical Manual of Mental Disorders, 3rd edition, revised (DSM-III-R), a literature review as well as the discussion of an expert panel. Fig. 1 shows the CAM diagnostic algorithm in a schematic way.

Since its introduction CAM-based delirium diagnosis has become the gold standard of delirium detection. Many subsequently developed assessment tools focused on inattention and acute onset/fluctuating course as core diagnostic criteria, as defined in the DSM‑5 in 2013 [3]. Also, the upcoming ICD-11 shifts its focus towards these criteria for delirium definition [33]. The assessment of other cognitive disturbances, such as logical thinking was skipped as it was too complex and less valid. Table 1 compares core diagnostic criteria and involved psychopathological findings of delirium according to ICD-10 and ICD-11 (Table 1; [26, 27]). The comparison shows how the definition of delirium has developed from a heterogeneous psychopathological syndrome to a more specific diagnosis based primary on temporal onset, vigilance and attention, so that the needs of acute and emergency care physicians in daily routine can be better addressed.

This review, therefore, presents a summary of the available evidence on the broad variety of delirium assessments and provides suggestions on delirium detection tools that might be particularly useful in an acute care setting.

## Methods

To identify the most relevant assessments, the Medline database was searched for reviews and validation articles of delirium screening instruments in older patients. Inclusion criteria were evaluation of assessment instruments in the context of delirium, English or German language and published between 2001 and 2021 (see supplementary material 1 for search string). These publications were screened for existing tests and the psychometric properties. In a second step, the cross-referenced literature of these reviews was examined in addition to the validation studies found to collect the primary literature of the respective assessments and extract further details. Thirdly, the Network for Investigation of Delirium: Unifying Scientists (NIDUS) website was used for an additional cross-reference search [41]. All instruments were investigated in detail by checking the full texts of the corresponding publications and extracting data on the number of study participants, investigators, scoring method, psychometric properties, time required for application and further aspects.

**Fig. 2:**
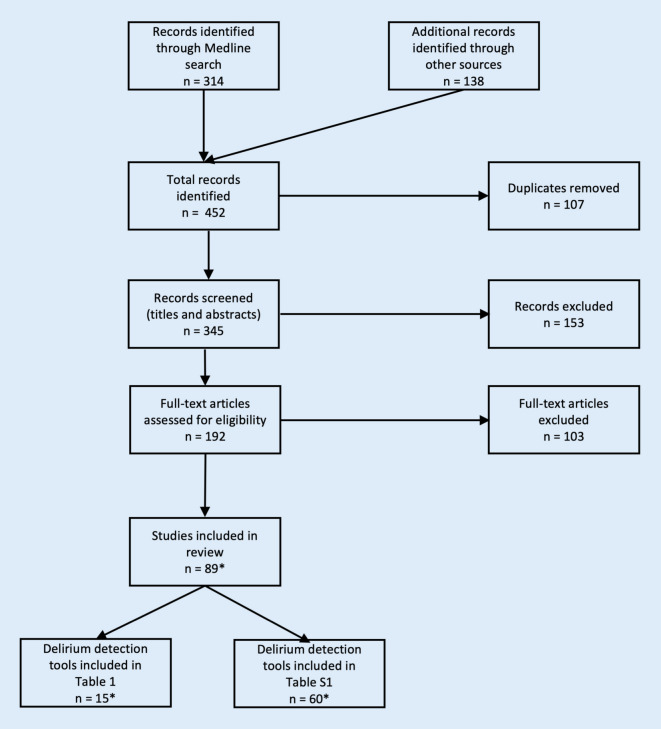
Study selection flow chart (*asterisk: *number of studies included and sum of identified delirium detection tools differ as for some tools data were extracted from two or more sources)

Fig. 2 illustrates the search, screening and selection process.

## Results

The search of Medline, cross-references and NIDUS website revealed 118 full-text articles that were evaluated in detail. A total of 75 delirium assessments were identified of which a selection of 15 tools with high clinical practicability and satisfactory psychometric properties are presented (Table 2). All other tools can be found in a separate table (Table S1) in the supplementary material.

**Table 2:** List of the 15 selected delirium detection tools and the corresponding characteristics

Delirium detection tools										
Abbreviation (full name)	Target patient group/investigator (I)	Screening vs. monitoring Items	Scoring	Average duration	Psychometric properties				Reference	Critical appraisal
					Sens	Spec	Others	RS		
Confusion assessment method (CAM) Family										
3D-CAM (3-minute diagnostic CAM)	General medicine patients ≥ 75 years (*n* = 201) (+ collateral history) I: trained physician/nurse	Screening 20 items (10 for patient interview, 10 observational) + 2 optional questions for collateral history All items referring to 4 core features 1. Acute onset and/or fluctuating course 2. Inattention 3. Disorganized thinking 4. Altered level of consciousness	CAM algorithm: 1 + 2 + (3 or 4) positive = suspected delirium	3 min	Total sample:		–	DSM-IV criteria	[38]	Good structure as guidance; operationalization of core features; less interviewer training required
					0.95	0.94				
					Patients with dementia:					
					0.96	0.86				
					Patients without dementia:					
					0.93	0.96				
CAM (short form)	Older patients ≥ 65 years (*n* = 56) + collateral history I: trained lay rater or clinician	Screening 4 items on core features (see 3D-CAM)	CAM algorithm: 1 + 2 + (3 or 4) positive = suspected delirium	5–10 min	0.94–1.00	0.90–0.95	IRR: presence/absence of delirium 100%, k = 1.0; assessing 4 core features 93%, k = 0.81	Geriatric psychiatrist rating after comprehensive assessment	[29]	Professional training required; poor sensitivity when CAM is conducted by untrained/insufficiently trained rater
CAM-ICU (CAM for the intensive care unit)	Adult ICU patients (*n* = 38) + collateral history I: trained lay rater or clinician	Screening 8 items on core features (see 3D-CAM)	CAM algorithm: 1 + 2 + (3 or 4) positive = suspected delirium	< 5 min	Total sample:		IRR: k = 0.95	DSM-IV criteria	[11]	Eligible for intubated ICU patients; operationalization of core features
					0.95–1.00	0.89–0.93				
					Patients ≥ 65 years:					
					0.90–1.00	0.83–1.00				
					Patients with dementia:					
					1.00	1.00				
FAM-CAM (Family CAM)	Community-dwelling older people ≥ 65 years with dementia (*n* = 52) Collateral history (caregiver/informant) I: trained rater/clinician	Screening 11 items	CAM algorithm: 1 + 2 + (3 or 4) positive = suspected delirium	5–10 min	0.88	0.98	Overall agreement with CAM 96%	CAM	[51]	Collateral history only
mCAM-ED (modified CAM for the emergency department)	ED patients ≥ 65 years (*n* = 286) (+ collateral history if available) I: trained clinician	Screening Two-step-rating: 1. MOTYB to identify inattention 2. If inattention present → MSQ for identifying cognitive impairment, Comprehension subtest of CTD for identifying disorganized thinking	Modified CAM algorithm: 1a (acute onset) AND 1b (fluctuating course) + 2 + (3 or 4) positive = diagnosed delirium, 1a OR 1b + 2 + (3 or 4) positive = suspected delirium	< 5 min	Total sample:		–	DSM-IV criteria	[18, 22]	Two-step approach; operationalization of core features
					0.90	0.98				
					Patients with dementia:					
					0.91	0.87				
					Patients without dementia:					
					0.89	0.99				
UB-CAM (Ultra-brief-CAM)	General medicine patients ≥ 75 years (*n* = 201) (+ collateral history) I: trained physician/nurse	Screening 2–15 items UB‑2, in the case of an incorrect answer followed by a modified 3D-CAM (assessment of each CAM feature is stopped after one incorrect answer or positive observation item of that feature)	CAM algorithm: 1 + 2 + (3 or 4) positive = suspected delirium	2 min	0.93	0.95	–	3D-CAM	[4, 25, 40]	Retrospective simulation based on 3D-CAM and UB‑2 data of two studies
*Other tools than CAM*										
4AT (4 As test)	Acute care and rehabilitation patients ≥ 70 years (*n* = 234) + collateral history I: untrained geriatrician	Screening 4 items: alertness AMT‑4 Attention (MOTYB) Acute change or fluctuation	Score 0–12 0 = no CI or delirium 1–3 = possible CI ≥ 4 = possible delirium	< 5 min	0.90	0.84	AUC 0.89–0.93	DSM-IV criteria	[5]	Information on acute change/fluctuation not mandatory for delirium diagnosis; no special training required; includes MOTYB
BCS (bedside confusion scale)	Palliative patients (*n* = 31) I: rater (no requirements)	Screening Tool 2 items: psychomotor activity + MOTYB	Score 0–5 Cut-off: ≥ 2 = suspected delirium	2 min	1.0	0.85	–	CAM	[52]	Includes MOTYB
DOS/DOSS (delirium observation screening scale)	Van Gemert: older patients ≥ 70 years (*n* = 87) Koster: cardiac surgery patients ≥ 45 years (*n* = 112) + collateral history (25-items version) I: nurse	Screening + Monitoring Severity scoring included Original version: 25 items Revised version: 13 items	Score 0–13 Final score = (score shift 1 + score shift 2 + score shift 3) / 3 Cut-off: ≥ 3 = suspected delirium	5 min (13-items version)	Van Gemert:		–	DSM-IV criteria	[17, 34, 49]	Suitable tool for periodical (once per shift) delirium screening by nurses
					0.89	0.88				
					Koster:					
					1.00	0.97				
DTS (delirium triage screen)	Adult patients (*n* = 406) I: trained clinician or lay rater	Screening 2 items	Scoring: First item (altered level of consciousness) positive = screening positive, specific delirium assessment required (recommended tool: bCAM). Negative first item is followed by second item (spelling “LUNCH” backwards), > 1 error = screening positive, specific delirium assessment required	< 1 min	DTS alone:		IRR: k = 0.79	DSM-IV-TR criteria	[20]	Two-step approach (combination with specific delirium assessment recommended)
					0.98	0.55				
					DTS + bCAM:					
					0.78–0.84	0.96–0.97				
MOTYB (months of the year backwards)	Adult patients, median age 69 years (*n* = 265) I: trained medical staff/nurse	Screening Months of the year forward, starting with January, followed by MOTYB, starting with December	At least “July” has to be reached without any error (omission or wrong month), error before July = suspected delirium	< 2 min	All participants (17–95 years):		–	DSM-IV criteria	[44]	Suitable as a quick and easy screening test for delirium Two-step approach recommended: positive result should be followed by specific delirium assessment
					0.83	0.91				
					Participants > 69 years:					
					0.84	0.90				
	ED patients ≥ 65 years (*n* = 286)	Screening MOTYB, starting with December, ending with January	1 point for each error; ≥ 8 points = suspected delirium		0.95	0.94	–	DSM-IV criteria	[23]	
		MOTYB, starting with December	All months named correctly at least from December to September September is not reached without error = suspected delirium		0.90	0.89				
mRASS (modified Richmond agitation sedation scale)	Older patients ≥ 65 years (*n* = 95) I: trained nurse	Screening + Monitoring Modified version of the RASS: Step 1: question “Describe how you are feeling today” Step 2: scoring the mRASS containing the additional aspect of attention	Screening: Any abnormal score (≠ 0) = suspected delirium Monitoring: any change to prior score	< 1 min	Used as a single instrument for delirium screening:		–	DSM-IV criteria	[7]	Suitable instrument to identify incident delirium (daily administration)
					0.64	0.93				
					Used as a monitoring instrument to detect change:					
					0.74	0.92				
Nu-DESC (nursing delirium screening scale)	Adult patients (*n* = 146) /nurse (who observed patient) I: trained clinician/nurse or lay rater	Screening + Monitoring Severity scoring included 5 items	Each item scored 0–2 (0 = absent, 1 = mild, 2 = severe) Total score 0–10 Cut-off: ≥ 2 = suspected delirium	< 2 min	0.86	0.87	–	CAM	[15, 16]	Suitable tool for periodical (once per day) delirium screening by nurses Subjective component of scoring a symptom “mild” or “severe”
SQiD (single question in delirium)	Patients with cancer, age 30–79 years (*n* = 21) Collateral history (friend/relative/caregiver) I: trained clinician or lay rater	One question: “Do you feel that … (patient’s name) has been more confused lately?”	Answer “yes” or “no” “Yes” = suspected delirium	< 1 min	Vs. psychiatrist interview				[48]	Suitable as a quick and easy delirium screening question for caregiver interview Two-step approach recommended: positive result should be followed by specific delirium assessment of patient
					0.80	0.71	–	–		
					Vs. CAM:					
					0.67	0.67	–	–		
					Vs. MMSE:					
					0.50	0.59	–	–		
	Patients ≥ 75 years (*n* = 100)				0.77	0.51	–	DSM-IV criteria	[36]	
UB‑2 (ultra-brief 2‑item screener)	Patients ≥ 75 years (*n* = 201) I: trained clinician	Screening 2 items: naming the current day of the week + MOTYB	Any incorrect answer, omission of one or more months or no answer/answer “I do not know” = suspected delirium	< 2 min	Sens 0.93	Spec 0.64	–	DSM-IV criteria	[13]	Suitable as a quick and easy screening test for delirium Two-step approach recommended: positive result should be followed by specific delirium assessment

*AMT-4* abbreviated mental test 4, *AUC* area under the curve, *CAM* confusion assessment method, *CI* cognitive impairment, *CTD* cognitive test for delirium, *DSM-IV* Diagnostic and Statistical Manual of Mental Disorders IV, *ED* emergency department, *ICC* intraclass correlation coefficient, *ICU* intensive care unit, *IRR* interrater reliability, *k* kappa, *MMSE* mini mental state examination, *MOTYB* months of the year backwards, *n* number, *RS* reference standard*, Sens* sensitivity, *Spec* specificity, *MSQ* mental status questionnaire

### The CAM family

A total of 13 tools belonging to the CAM family were identified, all focusing on the 4 core symptoms: acute onset and/or fluctuating course, inattention, disorganized thinking and altered level of consciousness.

All CAM derivatives share the underlying diagnostic algorithm but differ in their structure and targeted group of persons. Most instruments are designed for patient interviews supplemented by collateral history from relatives, staff and medical records, e.g. CAM, CAM-severity scale (CAM-S), brief CAM (bCAM) and CAM for the intensive care unit (CAM-ICU) [11, 20, 30]. They often include an informal interview by which the presence of the core symptoms is evaluated. An example for a clearly structured variant is the 3‑minute diagnostic CAM (3D-CAM) with 10 questions to be answered by the patient, 10 questions to be answered by the interviewer and 2 optional questions requiring collateral history provided by relatives, friends and/or caregivers and medical records examination. Other CAM instruments are based on patient observation, e.g. nursing home CAM (NH-CAM) or caregiver interview, e.g. family CAM (FAM-CAM) [9, 51]. Furthermore, several variants address specific subgroups of patients, such as the CAM-ICU, which is also applicable to intubated patients, or the CAM for the emergency department (CAM-ED) [10, 18].

The CAM and its derivatives have many advantages but also some disadvantages. Advantages are the short examination duration, the structured evaluation algorithm and the convincing results of numerous validation studies with sensitivity and specificity values of 90–100% for delirium diagnosis compared to extensive and time-consuming clinical diagnostics; however, an important disadvantageous aspect is that for correct application of most CAM instruments intensive training is required to ensure valid and reliable test results. Some studies demonstrated poor sensitivity when the CAM was conducted by untrained or insufficiently trained raters [47, 48]. For example, the symptom disorganized thinking requires an experienced assessor for its detection. This issue has been addressed by deriving the 3D-CAM with more operationalized features [38]. In future this limitation might be overcome as the new definitions of delirium according to DSM‑5 and ICD-11 no longer include the symptom of impaired thinking.

### Other delirium detection tools

For the emergency department and many other clinical settings there is a need for easy and quick screening tools. Examples for such assessments that could be integrated in daily clinical routine are the 4 As test (4AT) and the nursing delirium screening scale (Nu-DESC) [5, 15]. The 4AT has recently been evaluated against the CAM showing significant better sensitivity in a multicenter study [50].

As inattention is a characteristic delirium symptom many assessments that address this aspect have been evaluated in acute care and emergency settings [60]. The most popular of these is the months of the year backwards test (MOTYB) that provides a very quick option to screen for this feature. It is included in many assessments that combine testing for different aspects of delirium, such as the CAM. Several studies have evaluated its singular use in the context of delirium reporting high sensitivity and specificity [24]. Efforts have been made to further simplify the scoring method while maintaining good psychometric properties [23]. Another instrument, the bedside confusion scale (BCS), includes the MOTYB and adds one more question to assess a second delirium feature: psychomotor disturbance [52].

There are also easy to use tools focusing on the core feature acute onset. An example is the single question in delirium (SQID), which determines acute confusion by asking a relative or caregiver: “Do you feel that [patient’s name] has been more confused lately?” [48]. The SQiD can be used as a screening as well as a monitoring instrument for hospital staff.

An example for a quick tool examining the vigilance/level of consciousness of a patient, the change of which is another core feature of delirium, is the Richmond agitation sedation scale (RASS) [21]. It is widely used in intensive care units. The instrument assesses the vigilance on a scale from −5 (unarousable) to +4 (combative). There is a modified version, the mRASS, which has been optimized by adding the level of attention to each category [7].

There have been numerous attempts to use other tests originally designed for dementia diagnostics as delirium detection tools, for example the clock drawing test (CDT), but the studies revealed poor accuracy for this purpose, independent of the scoring method [1]. Other examples are the short portable mental status questionnaire (SPMSQ) or the abbreviated mental test (AMT), which are suitable to detect early dementia symptoms, but not to distinguish between dementia and delirium [8, 12].

While most of the identified tools serve solely as screening tools, some are also appropriate for monitoring the course of delirium. This group primarily comprises instruments including severity scoring and thus offering the possibility to observe change including the effect of measures taken. Examples are the CAM‑S, Nu-DESC or delirium observation screening scale (DOSS) [15, 30, 49].

## Discussion

Over the last decades different assessments supportive for delirium diagnosis have been developed. These assessments differ in length and structure, psychometric performance, screening vs. monitoring abilities and whether the patient or an informant is assessed. The main goal of a delirium screening tool should be the differentiation of delirium as a cause of cognitive impairment in an emergency/acute care setting. In this setting major differential diagnoses of delirium are dementia and delirium superimposed on dementia (DSD).

### Delirium vs. dementia vs. delirium superimposed on dementia

The focus on inattention most delirium assessments share seems feasible for different reasons: inattention is a typical feature of delirium but not dementia, and is easy and fast to evaluate in any acute care setting [14, 27]. In dementia inattention is typically not altered unless disease progresses to severe stages. Nevertheless, in non-Alzheimer’s dementias, such as Parkinson’s disease dementia, inattention might be a cognitive domain altered in earlier stages [6].

Acute change in cognitive function is another important feature to discriminate delirium from dementia. In contrast to delirium, dementia is characterized by a chronic progressive disease course. Therefore, a second focus of many delirium assessments is the documentation of acute onset and/or fluctuating course, a central feature of the CAM family; however, a main limitation is the necessity to obtain collateral history, particularly difficult in emergency situations.

Assessments like the UB-CAM or the delirium triage screen (DTS) address this limitation by combining a short screening with a focus on inattention, followed by a second assessment that includes collateral history [20, 40]. The 4AT, that enjoys growing popularity, contains the item acute onset/fluctuating course but its presence is not mandatory for positive delirium screening [50, 57].

In order to better discriminate delirium from DSD tools have been validated to address pre-existing cognitive decline by a structured collateral history. Good psychometric properties have been shown for the informant questionnaire on cognitive decline in the elderly (IQCODE) and the Alzheimer’s disease 8 (AD8) [14, 32].

### Psychomotor disturbances, delusions and hallucinations

Psychomotor change has been added to the CAM algorithm to improve specificity in discriminating delirium and dementia [55]. In addition, the combination of a letter recognition attention test with the observational scale of level of arousal (OSLA) improved test performance compared to both individual tests [46].

Further frequent psychopathologic findings found in delirium are delusions and hallucinations. They are usually not integrated into commonly used assessments because they lack sensitivity and specificity for delirium diagnosis although they often have therapeutic consequences. Antipsychotic treatment should, however, only be used for very distressing delusions and hallucinations [31].

### Additional diagnostics

Several other diagnostic techniques have been evaluated for delirium diagnostics. Electroencephalography (EEG) can visualize alterations in brain functioning that are common in delirium, and shows potential even in differentiating delirium from dementia [54]. Recently a study described a short 1‑min single-channel EEG with new automated power analysis as a potential delirium detection tool [42]. Besides this example most EEG studies need experienced staff to obtain valid results and are, therefore, currently not implemented on a large scale. Neuroimaging, such as functional magnetic resonance imaging (fMRI) that detects network disintegration, or fluorodeoxyglucose positron emission tomography (FDG-PET) that shows focal hypometabolism in the posterior cingulate cortex, an important region for attention, can elucidate further pathophysiology but are also costly and complex to administer [19, 39]. Actimetry or other digitalized assessments are still in their infancy; however, 14 of 16 patients with CAM-positive delirium in the Canadian PredicT study were able to use a tablet-based “serious game” tapping on targets, showing digital assessments as a feasible approach [35]. A multicenter proof of assessing inattention is pursued by the DelApp. This smartphone app tests attention by presentation and recall of a sequence of symbols [56]. Other smartphone applications offer digital versions of common interview-based assessment tools facilitating documentation and practicability of the assessments [4].

## Conclusion

Delirium is a common neuropsychiatric syndrome in geriatric patients especially in the context of emergency medicine and acute inpatient care. Its regular screening and monitoring should be mandatory. Many tailored delirium assessments with good psychometric properties for screening and monitoring have been adapted for emergency and acute care medicine needs.

Most of these assessments focus on inattention and acute onset/fluctuating course of disease in order to enable discrimination of delirium from dementia. The increasingly popular 4AT and several CAM-derived assessments include both items and can be used with confidence in different acute care settings. Imaging techniques and electronic assessments, such as mobile apps, are still not routinely available and feasible. Assessment selection should be driven by clinical setting as well as staff training, available time resources, feasibility and validity.

## Supplementary Information

Supplementary material 1: Search string in Medline (via PubMed) and Supplementary material 2: Table S1 with additional delirium detection tools

## Declarations

For this article no studies with human participants or animals were performed by any of the authors. All studies performed were in accordance with the ethical standards indicated in each case.
