# Erratum to: Comparison of delirium detection tools in acute care

**DOI:** 10.1007/s00391-022-02071-1

**Published:** 2022-05-18

**Authors:** Simone Brefka, Gerhard Wilhelm Eschweiler, Dhayana Dallmeier, Michael Denkinger, Christoph Leinert

**Affiliations:** 1 Institute for Geriatric Research, Ulm University Medical Center, Ulm, Germany; 2 Geriatric Center Ulm, Ulm, Germany; 3 Agaplesion Bethesda Hospital Ulm, Zollernring 26, 89073 Ulm, Germany; 4 Geriatric Center, University Hospital Tuebingen, Tuebingen, Germany; 5 University Hospital for Psychiatry and Psychotherapy Tuebingen, Tuebingen, Germany; 6 Dept. of Epidemiology, Boston University School of Public Health, Boston, USA

**Erratum to:**

**Z Gerontol Geriat 2021**

10.1007/s00391-021-02003-5

The authors would like to apologize for an error concerning the mCAM-ED and make the following corrections:

Page 109, Table 2, mCAM-ED

Column: Screening vs. monitoring/Items:

2. If inattention present → MSQ for identifying cognitive impairment, Comprehension subtest of CTD for identifying disorganized thinking

Column: Scoring

Modified CAM algorithm: 1a (acute onset) AND 1b (fluctuating course) + 2 + (3 or 4) positive = diagnosed delirium

1a OR 1b + 2 + (3 or 4) positive = suspected delirium.

Page 111, Legend table 2:

As a replacement for *SPMSQ*: *MSQ* mental status questionnaire.

