# Supplementary material for: Comparison of delirium detection tools in acute care: A rapid review
Source: Z Gerontol Geriatr. 2022 Jan 14;55(2):105–15. doi: 10.1007/s00391-021-02003-5 (PMC8921069; doi:10.1007/s00391-021-02003-5)
Supplement: Supplementary file 1 — Supplementary material 1: Search string in Medline (via PubMed) and Supplementary material 2: Table S1 with additional delirium detection tools [file 391_2021_2003_MOESM1_ESM.pdf]

Supplementary material for

Brefka, S., Eschweiler, G.W., Dallmeier, D., Denkinger, M., Leinert, C. Comparison of delirium detection tools in acute care. A rapid review. Z Gerontol Geriat 55 (2022). https://doi.org/10.1007/s00391-021-02003-5

Supplementary material 1: Search string in Medline (via Pubmed)

(((Delirium[MeSH Major Topic]) OR (Deliri*[Title/Abstract])) AND ((Diagnosis[MeSH Terms]) OR (Outcome Assessment (Health Care) [MeSH Terms]) OR (Patient Outcome Assessment[MeSH Terms]) OR (Geriatric Assessment[MeSH Terms]) OR (Diagnostic[Title/Abstract]) OR (diagnostical[Title/Abstract]) OR (Diagnosis[Title/Abstract]) OR (Assessment[Title/Abstract]) OR (Assessing[Title/Abstract]) OR (Test[Title/Abstract]) OR (testing[Title/Abstract]) OR (Tests[Title/Abstract]) OR (Screen[Title/Abstract]) OR (screening[Title/Abstract]) OR (validation[Title/Abstract]) OR ("Delirium Detection"[Title/Abstract])) AND (("Aged"[Mesh]) OR ("Frail Elderly"[Mesh]) OR ("Older person*"[Title/Abstract]) OR ("Older adult*"[Title/Abstract]) OR ("Older patient*"[Title/Abstract]) OR (Elder*[Title/Abstract]) OR ("Geriatric patient" [Title/Abstract]) OR (Geriatric*[Title/Abstract])) AND (("Hospitals"[Mesh]) OR ("Inpatients"[Mesh]) OR ("Hospitalization"[Mesh]) OR Hospital OR Hospitali*)) AND ((review[Filter] OR systematic review[Filter]) AND (english[Filter] OR german[Filter]) AND (aged[Filter] OR 80 and over[Filter])) AND ((review[Filter] OR systematic review[Filter]) AND (english[Filter] OR german[Filter]) AND (aged[Filter] OR 80andover[Filter]) AND (2001:2021[pdat]))

Supplementary material 2: Table S1

Table S1: Additional delirium detection tools

Abbreviation (full name)	Target patient group / investigator (I)	Screening vs. monitoring<br><br>Items	Scoring	Average duration	Psychometric properties	Reference	Critical appraisal
**CAM (Confusion Assessment Method) „Family“**							
bCAM (brief CAM)	ED patients ≥ 65 years (n = 406)<br>+ collateral history<br>I: trained rater/physician	Screening<br><br>7 items on the 4 core features:<br>1. Acute onset or fluctuating course<br>2. Inattention<br>3. Disorganized thinking<br>4. Altered level of consciousness (RASS)	CAM algorithm: 1 + 2 + [3 or 4] positive = suspected delirium	< 5 min	Sens 0.78-0.84<br>Spec 0.96-0.97<br>RS: delirium diagnosis by psychiatrist based on DSM-IV criteria<br>IRR: k=0.88	[21]	If no external information available and feature 2 + (3 or 4) positive, feature 1 is assumed positive

Table S1: Additional delirium detection tools							
**Abbreviation (full name)**	**Target patient group / investigator (I)**	**Screening vs. monitoring**<br><br>**Items**	**Scoring**	**Average duration**	**Psychometric properties**	**Reference**	**Critical appraisal**
CAM (long form)	Older patients ≥ 65 years (n = 56)<br>+ collateral history<br>I: clinician or lay rater	Screening<br><br>10 items (4 items on core features (see bCAM) + 6 additional items)	CAM algorithm: 1 + 2 + [3 or 4] positive = suspected delirium	5-10 min	Sens 0.94-1.00<br>Spec 0.90-0.95<br>RS: Geriatric psychiatrist rating after comprehensive assessment<br>IRR: presence/absence of delirium 100% k=1.0; rating all 10 clinical features 88% k=0.67; assessing 4 core features 93% k=0.81	[24]	Refers to most CAM tools: Professional training required; poor sensitivity when CAM is conducted by untrained/insufficiently trained raters
CAM-ED (Emergency Department)	ED patients ≥ 65 years (n = 35)<br>+ collateral history (medical records)<br>I: clinician or lay rater	Screening<br><br>10 items, 5 of which are core features:<br>1. Acute onset<br>2. Fluctuating course<br>3. Inattention<br>4. Disorganized thinking<br>5. Non-alertness	Modified CAM algorithm: 1. + 2. + 3. + [4. or 5.] positive (4/4) = delirium<br>[1 or 2] + 3 + [4 or 5] positive (3/4) = probable delirium<br>2/4 positive = possible delirium<br>≤ 1/4 positive = no delirium	5-10 min	No psychometric properties available (no validation study)<br><br>CAM-ED delirium diagnosis (38 of 385 screened patients) compared to delirium diagnosis by ED physician's customary evaluation: only 6 of 35 included delirious patients = 17 %	[33]	Additionally MMSE required
CAM-S (long form)	Sample 1: Surgical patients ≥ 70 years (n = 300)<br>Sample 2: General medicine patients ≥ 70 years (n = 919)<br>+ collateral history<br>I: trained rater/clinician	Screening + severity scoring<br><br>10 items (4 items on core features (see bCAM) + 6 additional items)<br><br>Acute onset or fluctuating course rated as absent (0) or present (1)<br>Other 9 items rated as absent (0), mild (1) or marked (2)	CAM algorithm: 1 + 2 + [3 or 4] positive = suspected delirium<br><br>Severity score 0-19, higher score = more severe delirium	10-15 min	IRR: ICC 0.88<br>Construct validity compared to Daily Confusion Rating r=0.80 in sample 1, r=0.64 in sample 2; compared to Brief Cognitive Screen r=0.72; compared to MMSE r=0.64<br>Predictive validity (Nursing home placement) RR=1.0, 1.4, 2.1, 2.5 RR=1.0, 1.4, 2.3, 3.9 across CAM-S long form severity levels, p-trend<0.001	[25]	Brief cognitive testing (e.g. SPMSQ) required
CAM-S (short form)	Surgical patients ≥ 70 years<br>(n = 300)<br>General medicine patients ≥ 70 years (n = 919)<br>+ collateral history<br>I: trained rater/clinician	Screening + severity scoring<br><br>4 items on core features (see bCAM)<br><br>Acute onset or fluctuating course rated as absent (0) or present (1)<br>Other 3 items rated as absent (0), mild (1) or marked (2)	CAM algorithm: 1 + 2 + [3 or 4] positive = suspected delirium<br><br>Severity score 0-7, higher score = more severe delirium	< 5 min	IRR: ICC 0.92<br>Construct validity compared to Daily Confusion Rating r=0.78 in sample 1, r=0.45 in sample 2; compared to Brief Cognitive Screen r=0.62; compared to MMSE r=0.41<br>Predictive validity (Nursing home placement) RR=1.0, 1.4, 2.1, 2.5 across CAM-S short form severity levels, p-trend<0.001	[25]	Brief cognitive testing (e.g. SPMSQ) required

Table S1: Additional delirium detection tools							
**Abbreviation (full name)**	**Target patient group / investigator (I)**	**Screening vs. monitoring**<br><br>**Items**	**Scoring**	**Average dura-tion**	**Psychometric properties**	**Refe-rence**	**Critical appraisal**
I-CAM	Older patients ≥ 80 years with dementia (n = 102)<br>+ collateral history<br>I: trained rater/clinician	Screening<br><br>CAM adapted to ICD-10 classification:<br>4 items on core features (see bCAM) + 1 item on psychomotor activity	CAM algorithm: 1 + 2 + [3 or 4] positive = suspected delirium<br><br>Suspected delirium + abnormal psychomotor activity = delirium<br><br>Suspected delirium + normal psychomotor activity → 2. I-CAM assessment after 24 hours<br><br>No suspected delirium + abnormal psychomotor activity → 2. I-CAM assessment after 24 hours	< 5 min	Psychomotor activity items added to all original CAM examinations:<br>Sens 0.55, Spec 0.97, AUC 0.96<br>Psychomotor activity items added sequentially only to original CAM examinations with "No suspected delirium"-result:<br>Sens 0.91, Spec 0.85, AUC 0.95<br>RS: delirium diagnosis by multidisciplinary consensus panel based on ICD-10 criteria	[60]	
NH-CAM (Nursing Home)	Nursing home residents (n = 35721)<br>Patient observation<br>I: nursing home staff	Screening<br><br>9 items on core features (see bCAM)<br><br>Use of modified items/variables included in the MDS-RAI	CAM algorithm: 1 + 2 + [3 or 4] positive = suspected delirium<br><br>2-3 items positive (but no delirium according to CAM algorithm) = subsyndromal delirium level 2<br><br>1 item positive = subsyndromal delirium level 1	5 min	No comparison to RS, only comparison to other MDS-based methods of delirium diagnosis using the same variables but other scoring algorithms<br>Predictive validity: NH-CAM delirium severity associated with mortality (HR 1.5-1.9) and rehospitalization (HR 1.1-1.3)	[10]	Use of MDS items as representatives of original CAM items partly imprecise (e.g. "mood decline over last 90 days as a proxy for "acute onset")<br>Information on HR only in abstract, not in fulltext
**Other tools than CAM**							
6-CIT (6-item Cognitive Impairment Test)	Patients ≥ 70 years (n = 470)<br>I: trained rater	Screening<br><br>6 items	Score 0-28<br><br>Cut-offs:<br>Overall: ≥ 9 = suspected delirium<br>No dementia: ≥ 8 = suspected delirium<br>Dementia: ≥ 12 = suspected delirium	< 5 min	Overall:<br>AUC 0.88, Sens 0.90, Spec 0.63<br>No dementia:<br>AUC 0.80, Sens 0.85, Spec 0.61<br>Dementia:<br>AUC 0.67, Sens 0.81, Spec 0.31<br>RS: delirium diagnosis based on DRS-R-98	[47]	Instrument identical with OMC (Orientation Memory Concentration Test)<br><br>Includes MOTYB
ALOC (Altered Level Of Conscious-ness)	General medicine patients ≥ 75 years (n = 201)<br>I: trained physician/nurse	Screening<br><br>1 item	Lethargy, stupor, coma, or hypervigilance present = suspected delirium	< 1 min	All patients:<br>Sens 0.19, Spec 0.99<br>Patients with dementia:<br>Sens 0.14<br>Patients without dementia/with MCI):<br>Sens 0.21	[14]	Clinical impression of consciousness is rated<br>Poor sensitivity in contrast to RASS (very similar approach)

Table S1: Additional delirium detection tools							
**Abbreviation (full name)**	**Target patient group / investigator (I)**	**Screening vs. monitoring**<br><br>**Items**	**Scoring**	**Average dura-tion**	**Psychometric properties**	**Refe-rence**	**Critical appraisal**
AMT (Abbreviated Mental Test)	Surgical patients ≥ 65 years<br>(n = 100)<br>I: trained (lay) rater	Screening + Monitoring of POD (postoperative delirium)<br>Preoperative AMT score required for comparison of results<br><br>10 items	Deterioration of ≥ 2 compared to preoperative score result = suspected delirium	< 5 min	Sens 0.93<br>Spec 0.84<br>RS: DAS (DSM-III criteria)	[44]<br>[35]	AMT as a single tool only investigated in surgical patients, “predelirious” AMT score required for comparison of test results<br>AMT is able to detect cognitive deficits, not delirium-typical characteristics
AMT-4	Acute stroke unit patients, median age 74 years (n = 111)<br>I: trained rater (medical student)	Screening<br><br>4 items	Score 0-4<br>Cut-off: < 4 = suspected delirium	< 2 min	Sens 0.83<br>Spec 0.61<br>RS: CAM	[30]	AMT-4 (like AMT) as a single tool is able to detect cognitive deficits, not delirium-typical characteristics
CAC-A (Clinical Assessment of Confusion - A)	Adult medical/surgical patients<br>(n = 129)<br>+ collateral history<br>I: trained lay rater/nurse	Screening Tool<br><br>25 items	Score 0-77<br>Severity scoring included<br>Cut-offs:<br>2-8 possible acute confusion<br>9-14 mild acute confusion<br>15-28 moderate acute confusion<br>> 28 severe acute confusion	5 min	Correlated with level of confusion from VAS-C; RS: none<br>PPV: 0.65<br>NPV: 0.79<br>IRR: r=0.88	[67]	Additionally MMSE and/or evaluation by psychogeriatric nurse recommended
CAC-B (Clinical Assessment of Confusion - B)	Adult medical/surgical patients<br>(n = 149)<br>+ collateral history<br>I: trained lay rater/nurse	Screening Tool<br><br>58 items	Severity scoring included<br>Cut-offs:<br>66-80 possible acute confusion<br>81-100 mild acute confusion<br>101-120 moderate acute confusion<br>> 120 severe acute confusion	10 min	Correlated with level of confusion from VAS-C; RS: none<br>Content Validity index: 0.88<br>Reliability: Cronbach’s alpha = 0.95<br>IRR: r=0.69	[16]	No access to reference<br>Additionally MMSE and/or evaluation by psychogeriatric nurse recommended
CCS/ADS (Communication Capacity Scale/Agitation Distress Scale)	Palliative patients (n = 30)<br>+ collateral history<br>I: nurse/clinical staff	Monitoring Tool<br><br>CCS 5: 5 items<br><br>ADS 6: 6 items	CCS 5:<br>Score 0-17<br><br>ADS 6:<br>Score 0-18<br><br>No cut-offs defined, higher scores indicate higher severity levels	5-10 min	IRR: Cronbach’s alpha coefficients = 0.91 CCS, 0.96 ADS<br>Validity: **CCS** compared to MDAS rho=0.78; compared to Sedation Scale rho=0.86; cognitive items on the MDAS and DRS rho=0.83; **ADS** compared to DRS rho=0.61; compared to agitation items on MDAS and DRS rho=0.61	[41]	Does not cover all aspects of delirium, not appropriate for screening

Table S1: Additional delirium detection tools							
**Abbreviation (full name)**	**Target patient group / investigator (I)**	**Screening vs. monitoring**<br><br>**Items**	**Scoring**	**Average dura-tion**	**Psychometric properties**	**Refe-rence**	**Critical appraisal**
CDT (Clock Drawing Test)	ED patients ≥ 65 years (n = 406)<br>I: trained rater	Screening<br><br>Three methods:<br>Clinical gestalt<br>CAMDEX version (Score 0-3)<br>Shulman version (Score 0-5)	Clinical gestalt: normal/abnormal<br>CAMDEX: 0 = no clock face drawn, 3 = perfect clock<br>Shulman: 0 = no clock face drawn, 5 = perfect clock	< 5 min	Abnormal gestalt:<br>Sens 0.94-0.96, Spec 0.40-0.44<br>CAMDEX < 3:<br>Sens 0.94-0.96, Spec 0.40-0.41<br>Shulman < 5:<br>Sens 1.00, Spec 0.20-0.25<br>RS: delirium diagnosis by psychiatrist based on DSM-IV criteria	[11]	Unsuitable tool for delirium detection
	Older patients ≥ 70 years (n = 59)	Screening<br><br>Score 1-10 (Fisher/Flowerdew version)	Higher score = more accurate performance		No significant effect of DRS (p=0.711), CAM positive (p=0.969) or CAM negative (p=0.568) on CDT score<br>Significant effect of MMSE (p<0.0001) on CDT score	[2]	
CHART-DEL (Chart Review on Delirium)	Geriatric patients (n = 919)<br>Chart review<br>I: trained clinical staff	Screening + Monitoring<br><br>Related to the CAM core features<br>7 items	Delirium diagnosis classification depending on recorded core symptoms: definite, probable, possible, uncertain	15-30 min	Sens 0.74<br>Spec: 0.83<br>RS: trained interviewer ratings for delirium based on the CAM<br>IRR: k=0.41	[26]	Potential misclassification<br>Not recommended for diagnostic purposes
Cog-4 (Cognitive status - 4)	Acute stroke unit patients, median age 74 years (n = 111)<br>I: trained rater (medical student)	Screening<br><br>4 items on consciousness (orientation, executive function), language, inattention	Score 0-9<br>Cut-off: > 0 = suspected delirium	< 3 min	Sens 0.70<br>Spec: 0.44<br>RS: CAM	[30]	Subset of the National Institute of Health Stroke Scale (NIHSS)<br>Poor specificity for delirium detection
CRS (Confusion Rating Scale)	Older hip fracture patients<br>(n = 169) / nurse (who observed patient)<br>I: trained lay rater or clinician	Screening tool<br><br>Severity scoring included<br><br>4 items	Score 0-8 (for each item 0=absent, 1=mild, 2=severe)<br>Cut-off > 0 (= evidence of confusion)	1-2 min	RS: None<br>IRR: 86.5%<br>Agreement with SPMSQ: 78-79%	[69]	The authors themselves label the test as “immature”<br>Has been further developed into the Nu-DESC
CSE (Confusional State Examination)	Older patients (n = 71)<br>+ collateral history<br>I: trained clinical staff	Monitoring<br><br>Severity scoring included<br><br>22 items: confusion score (12 items), associated symptoms (7 items), intensity and duration (3 items)	Each item scored 0-4<br><br>Confusion score:<br>< 25 = mild delirium<br>25-35 = moderate delirium<br>> 35 severe delirium	< 30 min	IRR (confusion score): Spearman rank order correlation coefficient 0.89; weighted kappa coefficient 0.58<br>Sens to change: Correlation of change in confusion score over three weeks of pharmacological treatment to change in Clinical Global Impression scale for Improvement r=0.75	[52]	Not suitable for delirium screening or diagnosis

Table S1: Additional delirium detection tools							
**Abbreviation (full name)**	**Target patient group / investigator (I)**	**Screening vs. monitoring**<br><br>**Items**	**Scoring**	**Average dura-tion**	**Psychometric properties**	**Refe-rence**	**Critical appraisal**
CTD (Cognitive test for Delirium)	ICU patients (n = 103)<br>I: trained lay rater or clinician	Screening tool, two alternate forms (A and B)<br><br>9 items	Score 0-30<br>Cut-off ≤ 18	10-15 min	IRR: coefficient alpha=0.87<br>High correlation with MMSE in patients with delirium (0.82) and dementia (0.81)<br>Sens 1.0<br>Spec 0.95<br>RS: delirium diagnosis by psychiatrist based on DSM-III-R criteria	[22]	Test cannot reliably distinguish delirium from severe dementia
DAS (Delirium Assessment Scale)	Older patients (n = 60 or 48, unclear)<br>+ collateral history (staff)<br>I: physician	Monitoring<br><br>Severity scoring included<br><br>10 items	Score 0-26	< 10 min	IRR: 0.66-0.99<br>Sens / Spec 0.80-0.90<br>RS: delirium diagnosis by geriatrician based on DSM-III criteria	[45]	Test may be suitable for monitoring delirium severity<br>Not able to differentiate delirium from dementia
DCT2 (timed Digit Cancellation Test)	Geriatric patients (n = 110)<br>I: physician	Screening	Within a time-limit of 45 seconds the patient has to cross out two predefined numbers on a matrix with 110 numbers from 0-9 (totally 20/110, 1 point per match, minus 0.22 points for every error)<br>Cut-off < 9 (cognitively impaired patients: < 8)	5 min	All patients (cut-off < 9):<br>Sens 0.94, Spec 0.87<br>Cognitively impaired patients (cut-off < 8):<br>Sens 0.78, Spec 0.76<br>RS: delirium diagnosis by geriatrician based on DSM-III-R criteria	[56]<br>[46]	Good vision required
DDS (Delirium Detection Score)	ICU patients (n = 1073)<br>I: clinical staff	Screening + monitoring<br><br>Severity scoring included	Score 0-56	10 min	Sens 0.69<br>Spec 0.75<br>RS: Ramsay Sedation Scale (RSS), Sedation Agitation Scale (SAS), clinical assessment<br>IRR: physician-physician 0.74<br>physician-nurse 0.76<br>nurse-nurse 0.64	[49]	Modified from Clinical Withdrawal Assessment for Alcohol – revised (ClWA-Ar)<br>Low sensitivity and specificity<br>Sedation scales and “clinical assessment” (no details) as RS

Table S1: Additional delirium detection tools							
**Abbreviation (full name)**	**Target patient group / investigator (I)**	**Screening vs. monitoring**<br><br>**Items**	**Scoring**	**Average dura-tion**	**Psychometric properties**	**Refe-rence**	**Critical appraisal**
DDT-Pro (Delirium Diagnostic Tool-provisional)	Kean:<br>Adult patients with acquired brain damage (n = 36)<br>Franco:<br>Internal medicine patients ≥ 60 years (n = 200)<br>+ collateral history<br>I: trained lay rater or clinician	Screening tool<br><br>3 items	Score 0-9<br><br>Cut-off 7 (< 7 = suspected delirium, ≥ 7 = no delirium)	5 min	Kean:<br>Sens 1.0, Spec 0.94<br>Franco:<br>Sens 0.88-0.90, Spec 0.81-0.85<br>RS: delirium diagnosis by psychiatrist based on DSM-5 criteria and DRS-R-98	[29]<br>[15]	According to authors (Franco et al.) "further validation warranted before widespread use can be recommended"
DelApp (Delirium App)	Geriatric and orthopedic patients ≥ 70 years (n = 156)<br>I: trained psychologist	Screening + Monitoring<br><br>Arousal assessment (patient awake and able to follow commands?), followed by attention task (counting stars on smartphone display while distracting triangle shapes appear around stars)	Score 0-10<br>(Arousal assessment 0-4, attention task 0-6)<br><br>Cut-off: ≤ 8 = suspected delirium	< 5 min	All patients:<br>Sens 0.98, Spec 0.93 (cut-off ≤ 8)<br>Delirium vs. dementia:<br>Sens 0.98, Spec 0.87 (cut-off ≤ 8)<br>RS: delirium diagnosis based on CAM and DRS-R-98	[61]	Second study shows poorer sensitivity and specificity<br>According to authors further studies needed
	General/acute medical hospital patients ≥ 65 years (n = 187)<br>I: trained psychologist				All patients:<br>Sens 0.92, Spec 0.74 (cut-off ≤ 8)<br>Delirium vs. dementia:<br>Sens 0.92, Spec 0.52 (cut-off ≤ 8)<br>Sens 0.82, Spec 0.68 (cut-off ≤ 6)<br>RS: neuropsychological test battery based on DSM-5 criteria	[62]	
DI (Delirium Index)	Older adults ≥ 65 years (n = 318)<br>I: trained lay rater or clinician	Severity scoring tool<br><br>7 items	Score 0-21 (higher score = higher severity of delirium)	10 min	IRR: ICC 0.98<br>Convergent validity compared to MMSE:<br>Spearman correlation coefficient<br>delirium and dementia patients -0.83<br>delirium patients only -0.79<br>dementia patients only -0.78<br>neither delirium nor dementia -0.66	[36]	Has to be combined with MMSE (at least questions 1-5) or other cognition test<br>Suitable for scoring and monitoring of delirium severity, not for delirium screening

Table S1: Additional delirium detection tools							
**Abbreviation (full name)**	**Target patient group / investigator (I)**	**Screening vs. monitoring**<br><br>**Items**	**Scoring**	**Average dura-tion**	**Psychometric properties**	**Refe-rence**	**Critical appraisal**
DMSS (Delirium Motor Subtype Scale)	Meagher:<br>Palliative patients (n = 152)<br>Grover:<br>Adult psychiatric patients (n = 160)<br>Garcia Nuñez:<br>Surgical ICU patients (n = 289)<br>I: trained clinical staff	Identification of motor subtypes (hyperactive, hypoactive, mixed, no motor subtype)<br><br>Two versions with 11 items (Meagher 2008) / 13 items (Grover 2013): 4 (5) items for hyperactivity, 7 (8) items for hypoactivity	Cut-off: 2 “positive” items (hypoactivity: at least one of “decreased amount of activity” or “decreased speed of actions”)	10 min	DMSS-German (Garcia Nuñez):<br>IRR: Fleiss κ = 0.83<br>Motor subtypes (hyperactive, hypoactive, or mixed):<br>Sens 0.60-0.97<br>No motor subtype:<br>Sens 0.22<br>Spec for all individual subtypes 0.82-1.00<br>RS: delirium diagnosis by psychiatrist based on DSM-IV criteria and DRS-R-98	[38]<br>[19]<br>[17]	Not for delirium screening or monitoring, only suitable for determination of motor subtype
DMSS-4	Palliative patients, adult psychiatric patients (n = 487)<br>I: trained clinical staff	Identification of motor subtypes<br><br>Short version of the DMSS with 4 items: 2 hyperactive items, 2 hypoactive items	Cut-off: 1 “positive” item	< 5 min	Concordance for subtype attribution between original DMSS and DMSS-4: κ = 0.63	[37]	Not for delirium screening or monitoring, only suitable for determination of motor subtype
DOM (Delirium-O-Meter)	Older patients (n = 92)<br>I: clinical staff	Monitoring<br><br>Severity scoring included<br><br>12 items	Score 0-36 (higher score = higher delirium severity)	3-5 min	IRR on item level: 0.40-0.97, $p<0.05$.<br>Sens to change (DOM and DRS-R-98 each assessed at 3 time points, comparison of ΔDOM and ΔDRS-R-98): rho=0.80-0.95, $p<0.001$	[27]	Suitable for scoring and monitoring of delirium severity, not for delirium screening
DRS (Delirium Rating Scale)	Rockwood:<br>Geriatric/psychiatric patients<br>(n = 791)<br>Rosen:<br>Geriatric/psychiatric patients<br>(n = 104)<br>+ collateral history<br>I: trained clinical staff	Screening + monitoring<br><br>Severity scoring included<br><br>10 items	Score 0-32<br><br>Cut-off ≥12/≥10/≥7.5	Estimated time: Scoring: 15 min Gathering information from family, staff, medical records: variably	IRR: ICC 0.91-0.97<br>Sens 0.82-0.94<br>Spec 0.82-0.94 (with cut-off ≥10)<br>RS: delirium diagnosis by psychiatrist based on DSM-III criteria	[65]<br>[64]<br>[53, 54]	Cut-off inconsistent: Original article: no information; Trzepacz 1999: “about 12 points”; some studies (Rockwood 1996, Rosen 1994) used other cut-offs (10 p., 7.5 p.)

Table S1: Additional delirium detection tools							
**Abbreviation (full name)**	**Target patient group / investigator (I)**	**Screening vs. monitoring**<br><br>**Items**	**Scoring**	**Average dura-tion**	**Psychometric properties**	**Refe-rence**	**Critical appraisal**
DRS-R-98 (Delirium Rating Scale-Revised-98)	Adult patients (n = 68)<br>+ collateral history<br>I: trained clinical staff	Screening + monitoring<br><br>Severity scoring included<br><br>16 items (13 severity items, 3 diagnostic items)	Total score 0-46<br><br>Cut-off ≥ 17.75<br><br>Severity score 0-39<br><br>Cut-off ≥ 15.25	Scoring: 20-30 min<br>Gathering information from family, staff, medical records: 1-2 hours	Total score:<br>Sens 0.91-1.00, Spec 0-85-1.00<br>Severity score:<br>Sens 0.86-1.00, Spec 0.77-0.93<br>RS: DRS, CTD, Clinical Global Impression (CGI), all rated by psychiatrist<br>IRR: ICC total score 0.98; ICC severity score 0.99	[66]	Time-intensive tool, and for that reason limited clinical applicability<br>Suitable for use in longitudinal studies on delirium, e.g. treatment research
DSB (Digit Span Backwards)	Leung:<br>Older (≥75 years) acute admission patients (n = 144)<br>O'Keeffe:<br>Acute geriatric patients (n = 110)<br>I: trained clinical staff	Screening + Monitoring (with other digits)	Cut-off <3 (Leung) /<br><4 (O'Keeffe)	5 min	Leung:<br>Sens 0.81, Spec 0.63<br>O'Keeffe:<br>Sens 0.83, Spec 0.96<br>RS: delirium diagnosis by geriatrician based on DSM-III-R criteria	[32]<br>[46]	Effective to identify patients with major cognitive impairment<br>Not suitable as a single tool in delirium diagnosis
DSI (Delirium Symptom Interview)	Older acute care patients ≥65 years (n = 50)<br>I: trained clinician or lay rater	Screening + Monitoring<br><br>32 items representing 7 domains	Disorientation **or** disturbance of consciousness **or** perceptual disturbance classified as "present" = suspected delirium	10-15 min	IRR: k=0.90<br>Sens/Spec 0.90/0.80<br>RS: delirium diagnosis by psychiatrist/neurologist	[3]	Extensive instrument with large number of items, thus time-intensive and of limited use for everyday clinical practice

**Table S1: Additional delirium detection tools**							
**Abbreviation (full name)**	**Target patient group / investigator (I)**	**Screening vs. monitoring**<br><br>**Items**	**Scoring**	**Average dura-tion**	**Psychometric properties**	**Refe-rence**	**Critical appraisal**
DSS (Delirium Severity Scale)	Acute care patients ≥ 55 years without dementia (n = 37)<br>I: trained clinician or lay rater	Monitoring<br><br>Modified versions of Forward Digit Span of the Wechsler Memory Scale–Revised (WMS-R) + Similarities (WAIS-R/WISC-R)<br>Modifications: addition of forward spans with two digits and one digit in cases of fails on the three-digits spans, addition of a third trial at each span length; combination of items of the Wechsler Adult Intelligence Scale-Revised (WAIS-R) and the Wechsler Intelligence Scale for Children-Revised (WISC-R)	Forward Digit Span: 0-24<br>Similarities: 0-35<br><br>No information on cut-offs for different severity categories	10 min	IRR: ICC = 0.99<br>Concurrent validity (Correlation with quantitative geriatric psychiatrist ratings of delirium severity): r=-0.44 to -0.52 (p=0.02/0.05)	[5]<br>[9]	Taking account of test acceptability to delirious patients<br>Fee-required test components<br>Not applicable to demented subjects
GAR (Global Attentiveness Rating)	Geriatric patients (n = 87)<br>I: physician (trained clinician)	Screening + Monitoring<br>Method: general conversation between physician and patient for a minimum of 2 minutes	Scoring of attentiveness by physician on a visual analog scale (0-10 cm), answering the question: How well did the patient keep his mind on interacting with you during the interview?<br>Score 0-10<br>Cut-off: < 7 = suspected delirium	2 min (minimum)	IRR: ICC 0.83<br>All patients (with and without cognitive impairment):<br>Sens: 0.94, Spec: 0.99<br>Cognitively impaired patients:<br>Sens: 0.94, Spec: 0.94<br>RS: delirium diagnosis by geriatrician based on DSM-III-R criteria	[46]	Much experience with delirious patients required, no objective criteria available, subjective rating
GCS (Glasgow Coma Scale)	Acute stroke unit patients, median age 74 years (n = 111)<br>I: trained rater (medical student)	Screening	Score: Eye opening (E) 1-4, Verbal response (V) 1-5<br>Cut-off: E < 4 +/- V < 5 = suspected delirium	< 1 min	Sens 0.17<br>Spec 0.81<br>RS: delirium diagnosis based on CAM	[30]	Attempt to use GCS for delirium screening, results (poor sensitivity) similar to ALOC results RASS/mRASS more suitable
I-AGeD (Informant Assessment of Geriatric Delirium)	Geriatric patients with caregiver<br>(n = 88)<br>Observation of caregiver (self-administration of questionnaire by caregiver)	Screening<br><br>10 items	Score 0-10<br>Cut-off: > 4 = suspected delirium	5 min	Sens/Spec compared to CAM:<br>0.82/0.64<br>Sens/Spec compared to clinical diagnosis of delirium by geriatricians using DSM-IV criteria:<br>0.70-0.89/0.63-1.00	[50]	Missing data as limitation

**Table S1: Additional delirium detection tools**							
**Abbreviation (full name)**	**Target patient group / investigator (I)**	**Screening vs. monitoring**<br><br>**Items**	**Scoring**	**Average dura-tion**	**Psychometric properties**	**Refe-rence**	**Critical appraisal**
ICDSC (Intensive Care Delirium Screening Checklist)	ICU patients (n = 93)<br>+ collateral history<br>I: clinician/nurse	Screening<br><br>8 items	Score 0-8<br>Cut-off: ≥ 4 = suspected delirium<br>1-3 = subsyndromal delirium	< 5 min	Sens/Spec (estimated from ROC curve, at cut-off score of 4 points):<br>0.99/0.64<br>RS: delirium diagnosis by psychiatrist	[4, 71]	Utility as a screening tool, not as a diagnostic tool (because of low specificity)
Inter-RAI AC (Inter Resident Assessment Instrument Acute Care) Delirium Screener	Acute care patients ≥ 70 years<br>(n = 239)<br>+ collateral history<br>I: trained nurse	Screening<br><br>2 items (acute change and fluctuation of mental function = 2 of 4 delirium items contained in the Inter-RAI AC Assessment System consisting of a total of 62 items)	1 of 2 items positive = suspected delirium	< 5 min	Sens 0.82<br>Spec 0.91<br>RS: clinical delirium diagnosis by experienced geriatrician, based on DSM-IV criteria	[57]	In the context of Inter-RAI AC use tool seems suitable for delirium screening
IPT (Interlocking Pentagons Test)	Geriatric medical patients, mean-age 79.9 years (n = 193)<br>I: trained rater	Screening<br><br>Figure of two intersecting pentagons has to be copied	Score 1-6<br>Cut-off: < 4 = suspected delirium	< 2 min	Sens 0.71<br>Spec 0.73<br>RS: delirium diagnosis based on DSM-IV criteria and DRS-R-98	[31]	Test is part of MMSE, able to detect cognitive deficits, not delirium-typical characteristics
MDAS (Memorial Delirium Assessment Scale)	Patients with cancer or AIDS<br>(n = 33)<br>+ collateral history<br>I: trained psychiatrist/ psychologist	Screening + Monitoring<br><br>Severity scoring included<br><br>10 items	Each item scored 0-3<br>Total score 0-30<br>Cut-off: ≥ 13 = suspected delirium	≥ 10 min patient assessment + 15-30 min external assessment	IRR: ICC = 0.92 (2 psychiatrists)<br>Sens/Spec 0.71/0.94	[7]	Time-intensive, not suitable for every-day clinical use
NEECHAM (Neelon and Champagne) Confusion Scale	Neelon:<br>Patients ≥ 65 years<br>(n = 158, pilot testing)<br>+ collateral history (medical records)<br>I: trained clinician or lay rater	Screening + Monitoring<br><br>Severity scoring included<br><br>9 items in 3 subscales: processing, behavior, physiological control	Each item scored 0-2, 0-4, or 0-5<br><br>Total score 0-30<br>(Processing score 0-14, behavior score 0-10, physiological control score 0-6)<br><br>30-27=non-delirious<br>26-25=at risk<br>24-20=early to mild confusion<br>19-0=moderate to severe confusion	8-10 min	Pilot testing (n = 158):<br>Sens 0.95, Spec 0.78	[43]<br>[18]	Authors name acute onset and fluctuating course as core symptoms of delirium, but neither of these two aspects is integrated in the NEECHAM instrument

Table S1: Additional delirium detection tools

Abbreviation (full name)	Target patient group / investigator (I)	Screening vs. monitoring<br><br>Items	Scoring	Average dura-tion	Psychometric properties	Refe-rence	Critical appraisal
OBS (Organic Brain Syndrome Scale)	Cardiac surgical patients ≥ 60 years (n = 52)<br>I: trained rater	Screening<br><br>Disorientation subscale 16 items<br>Confusion subscale 39 items	Score 0-165 (each item 0-3)<br><br>No cut-off defined	10-15 min	"Complete agreement with CAM" concerning delirium diagnosis<br>Items addressing 4 domains significantly differentiating delirious from non-delirious patients: concentration, disorientation, memory, latency in reaction and response to verbal stimuli	[6]<br>[12]	Insufficient information on psychometric properties<br>Small sample<br>Tool seems to be widely used in Scandinavia, often as the reference standard for delirium diagnosis
OMC (Orientation Memory Concentration Test)<br>→ see 6-ICT	see 6-ICT	see 6-ICT	see 6-ICT	see 6-ICT	see 6-ICT	[28]	
OSLA (Observational Scale of Level of Arousal)	Acute and rehabilitation hospital patients ≥ 70 years (n = 114)<br>I: "delirium expert"/physician	Screening + Monitoring<br><br>5 items: eye opening, eye contact, posture, movement, communication	Score 0-19<br><br>Cut-off: ≥ 4 = suspected delirium	< 1 min	All patients:<br>Sens 0.85, Spec 0.92, AUC: 0.92<br>Patients with dementia:<br>Sens 0.74, Spec 0.96, AUC: 0.93<br>RS: delirium diagnosis by physician based on DSM-5 criteria	[51]	Small sample size per site<br>No information on blinding of reference standard and delirium detection tool assessors
OSLA/SAVEAHA ART	Acute and rehabilitation hospital patients ≥ 70 years (n = 109)<br>I: "delirium expert"/physician	Screening<br><br>Combination of the tools OSLA and SAVEAHAART	Score 0-29<br><br>Cut-off: ≥ 10 = suspected delirium	< 2 min	All patients:<br>Sens 0.84, Spec 0.92, AUC 0.94<br>Patients with dementia:<br>Sens 0.94, Spec 0.92, AUC 0.98<br>RS: delirium diagnosis by physician based on DSM-5 criteria	[51]	Small sample size per site<br>No information on blinding of reference standard and delirium detection tool assessors
O3DY (Ottawa 3DY)	ED patients ≥ 65 years (n = 301)<br>I: trained rater	Screening<br><br>Serial administration twice daily (at least 6 hours between measurements), starting after 8 hours ED stay and ongoing up to 24 hours after hospital ward admission<br><br>4 items	Any incorrect answer/error = cognitive dysfunction/ suspected delirium	< 5 min	Patients with at least one positive O3DY compared to patients with at least one positive CAM:<br>Sens 0.87, Spec 0.44<br>O3DY result compared to CAM result in same patient assessment:<br>Sens 0.63, Spec 0.66<br>RS: delirium diagnosis based on CAM	[70]	Only patients without significant cognitive impairment included

Table S1: Additional delirium detection tools							
**Abbreviation (full name)**	**Target patient group / investigator (I)**	**Screening vs. monitoring**<br><br>**Items**	**Scoring**	**Average dura-tion**	**Psychometric properties**	**Refe-rence**	**Critical appraisal**
RADAR (Recognizing Acute Delirium As part of your Routine)	Patients ≥ 65 years (n = 193)<br>I: trained clinical staff or lay rater	Screening<br><br>3 items to score while giving patients their medication: drowsiness, problems with following instructions, slow movements	Each item rated "yes" or "no".<br><br>Cut-off: ≥ 1 "yes" = suspected delirium	< 1 min	IRR of instrument items: Agreement 82.4-98.0% (k=0.34-0.79)<br>Convergent validity (RADAR item compared to corresponding CAM item): Agreement 52-85%<br>Concurrent validity (RADAR item compared to DSM-IV-TR criterion): Sens 73%, Spec 67%	[68]	Moderate to low psychometric properties<br>Advantage: potential for routine use
RASS (Richmond Agitation Sedation Scale)	ED patients ≥ 65 years (n = 406)<br>I: trained rater/physician	Screening + Monitoring	RASS > 0 or < 0<br>(RASS > 1 or < -1)	< 1 min	RASS > 0 or < 0:<br>Sens 0.82-0.84, Spec 0.85-0.88<br>RASS > 1 or <-1:<br>LR+ 19.6-57.0<br>RS: delirium diagnosis based on DSM-IV criteria<br>IRR: weighted k=0.63	[20]	mRASS with better specificity but interestingly lower sensitivity<br>Compared to ALOC and GCS (similar approach) higher sensitivity
RCDS (Reversible Cognitive Dysfunction Scale)	Patients ≥ 65 years (n = 80)<br>I: trained clinical staff	Screening<br><br>Severity scoring included<br><br>6 items + MMSE score	Items 1-4 each scored 0-4<br>Item 5 scored 0-3<br>Item 6 scored 0-5<br><br>Sum of six item scores minus MMSE score plus 30 = total score (score 0-54)<br><br>Cut-off: ≥ 16 = suspected delirium	5 min	PPV/NPV (compared to operationalized reversible cognitive dysfunction): 92.3/96.7<br>Convergent validity:<br>Agreement with DSM-III-R k=0.69<br>Agreement with ICD-10 k=0.69<br>Agreement with CAMDEX k=0.83<br>Agreement with DRS k=0.60<br>Agreement with CAM k=0.45	[63]	Additional MMSE application required
SAVEAHAART	Acute and rehabilitation hospital patients ≥ 70 years (n = 109)<br>I: "delirium expert"/physician	Screening<br><br>"S-A-V-E-A-H-A-A-R-T" (10 single letters) is read, of which 4 letters are "A", patient has to indicate each time an "A" is heard	Score 0-10<br>Cut-off:<br>All patients: ≥ 4 errors (incorrect or omitted indication) = suspected delirium<br>Patients with dementia: ≥ 7 errors (incorrect or omitted indication) = suspected delirium	< 1 min	All patients:<br>Sens 0.90, Spec 0.64, AUC 0.80<br>Patients with dementia:<br>Sens 0.84, Spec 0.73, AUC: 0.79<br>RS: delirium diagnosis by physician based on DSM-5 criteria	[51]	Small sample size per site<br>No information on blinding of reference standard and delirium detection tool assessors<br>Defined cut-offs differ substantially from the VAT cut-off
SDC (Saskatoon Delirium Checklist)	Presurgical patients (n = 36)<br>I: trained clinician or lay rater	Screening<br><br>10 items (DSM-III criteria)	Each item scored 0-4<br>Total score 0-40 (40 = unimpaired, 0 = maximal delirium)<br><br>Cut-off: not available	< 5 min	Not available	[39]	No definition/ operationalization of symptoms, cut-off value, psychometric properties available

Table S1: Additional delirium detection tools							
**Abbreviation (full name)**	**Target patient group / investigator (I)**	**Screening vs. monitoring**<br><br>**Items**	**Scoring**	**Average dura-tion**	**Psychometric properties**	**Refe-rence**	**Critical appraisal**
Sour Seven Questionnaire	Medical and orthopedic inpatients ≥ 65 years (n = 39)<br>I: observation of untrained caregiver (family member/nurse), questionnaire administration (caregiver) by medical student	Screening/Diagnosis<br><br>7 items	Score 0-18<br><br>Cut-off:<br>≥ 4 possible delirium<br>≥ 9 being diagnostic for delirium	2-5 min	Sens 0.90, Spec 0.90 (cut-off ≥ 4)<br>Sens 0.63, Spec 1.00, PPV 1.00 (cut-off ≥ 9)<br>RS: delirium diagnosis based on CAM/assessment by geriatric psychiatrist based on DSM-5 criteria	[58]	Pilot study, further evaluation needed<br>High acceptance of the questionnaire by caregivers/nurses
SPMSQ (Short Portable Mental Status Questionnaire)	Medical inpatients ≥ 65 years<br>(n = 262)<br>I: psychologist/research assistant	Screening<br><br>10 items	Score 0-10 (number of errors)<br><br>Cut-off: ≥ 3 = suspected delirium	< 3 min	Sens 0.73<br>Spec 0.89<br>RS: delirium diagnosis based on "standard clinical criteria"	[13]<br>[40]	No information on blinding of reference standard and SPMSQ assessors<br>According to authors instrument not very suitable for delirium screening
SQeeC (Simple Question for easy evaluation of Consciousness)	Patients ≥ 75 years (n = 100)<br>I: trained clinician or lay rater	Two questions:<br>1."Name a place you would like to visit that you have never been before" (modified version: "Imagine that you are well, name a place you would like to visit that you have never been before")<br>2. "How would you make the journey?"	Patient can't name a place and/or can't provide a logical mode of transport to get there = suspected delirium	< 1 min	All patients:<br>Sens 0.83, Spec 0.81<br>Subgroup analysis in dementia patients:<br>Sens 0.83, Spec 0.59<br>RS: clinical delirium diagnosis by experienced geriatrician, based on DSM-IV criteria	[34]	According to subgroup analysis not suitable for distinguishing delirium from dementia
SSF (Spatial Span Forwards)	General hospital adult inpatients<br>(n = 265)<br>I: trained junior medical staff	Screening<br><br>Sequences of tapped out squares (in total 8 red squares on a white A5-sized card) have to be repeated, starting with a sequence of 2, maximum sequence being 7	Last correctly repeated sequence ≤ 4 = inattention (suspected delirium)<br>or<br>Last correctly repeated sequence ≤ 3 = inattention (suspected delirium)	< 5 min	Cut-off ≤ 4:<br>Sens 0.92, Spec 0.69<br>Cut-off ≤ 3:<br>Sens 0.77, Spec 0.86<br>RS: delirium diagnosis by experienced psychiatrist, based on DRS-R-98 and DSM-IV criteria	[48]	Depending on cut-off, either sensitivity or specificity are rather low<br>Might be useful as a single tool in patients without preexisting dementia
SSQ-Delirium (Single Screening Question-Delirium)	Caregivers of acute geriatric inpatients (n = 70)<br>I: self-administration by caregiver	Screening<br><br>Question to caregiver: "How has your relative's/friend's memory changed with his/her current illness?"	Score 1-5 (1 = much improved, 5 = much worse)<br><br>Cut-off: ≥ 4 = suspected delirium	< 1 min	Sens 0.77<br>Spec 0.56<br>RS: delirium diagnosis based on CAM conducted by trained senior medical student	[23, 55]	Moderate sensitivity, low specificity<br>Advantage: no specific training required, easy-to-use tool

**Table S1: Additional delirium detection tools**							
**Abbreviation (full name)**	**Target patient group / investigator (I)**	**Screening vs. monitoring**<br><br>**Items**	**Scoring**	**Average dura-tion**	**Psychometric properties**	**Refe-rence**	**Critical appraisal**
VAS-AC (Visual Analog Scale for Acute Confusion)	Long term care residents (n = 74)<br>I: trained clinician or lay rater	Screening<br><br>2 items (?)	Line of 100 mm length<br>0 mm point = no acute confusion<br>1-9 mm = possible acute confusion<br>10-39 mm = mild acute confusion<br>40-69 mm = moderate acute confusion<br>70-100 mm = severe acute confusion	< 5 min	IRR = 0.80<br>Concurrent validity correlated with DSM-IV criteria: Pearson's r = –0.81 (p < 0.001)<br>Sens 0.97<br>Spec 0.81	[8, 42]	Derived from the VAS-C (Visual Analog Scale for Confusion) by adding an "A" for "acute"<br><br>No access to original article (Nagley 1986), so items/ question wording could not be determined
VAT (Vigilance "A" Test)	Acute care patients ≥ 70 years<br>(n = 200)<br>I: trained rater	Screening<br><br>List of 29 letters is read, of which 11 letters are "A", patient has to indicate each time an "A" is heard	Cut-off: > 2 errors (incorrect or omitted indication) = suspected delirium	< 5 min	Sens 0.82<br>Spec 0.60<br>RS: delirium diagnosis based on CAM	[1]<br>[59]	Different Versions of VAT available, one other version consisting of 60 letters with 18 "A"<br>Error-free performance indicates adequate attention, but failure may reflect different limitations (also others than attention)
WORLD Backwards Test	Geriatric medical patients, mean-age 79.9 years (n = 193)<br>I: trained rater	Screening<br><br>5 letters to be spelled backwards, 1 point for each correct letter	Score 0-5<br>Cut-off: < 5 = suspected delirium	< 2 min	Sens 0.90<br>Spec 0.41<br>RS: delirium diagnosis based on DSM-IV criteria and DRS-R-98	[31]	Test is part of MMSE, able to detect cognitive deficits, not delirium-typical characteristics

AIDS *acquired immune deficiency syndrome*, AUC *area under the curve*, CAM *confusion assessment method*, CAMDEX *Cambridge mental disorders of the elderly examination*, DAS *delirium assessment scale*, DSM *Diagnostic and Statistical Manual of Mental Disorders*, ED *emergency department*, HR *hazard ratio*, I *investigator*, ICC *intraclass correlation coefficient*, ICD-10 *International Statistical Classification of Diseases and Related Health Problems 10*, ICU *intensive care unit*, IRR *interrater reliability*, k *kappa*, LR+ *positive likelihood ratio*, MDS *minimum data set*, MMSE *mini mental state examination*, n *number*, NPV *negative predictive value*, p *probability*, PPV *positive predictive value*, r *correlation coefficient*, RAI *resident assessment instrument*, RASS *Richmond agitation sedation scale*, ROC *receiver operating characteristic*, RR *risk ratio*, RS *reference standard*, Sens *sensitivity*, Spec *specificity,* SPMSQ *short portable mental status questionnaire*, VAS-C *visual analog scale for confusion*

References Table S1

1. Adamis D, Meagher D, Murray O et al (2016) Evaluating attention in delirium: A comparison of bedside tests of attention. Geriatr Gerontol Int 16:1028–1035. https://doi.org/10.1111/ggi.12592

2. Adamis D, Morrison C, Treloar A et al (2005) The Performance of the Clock Drawing Test in Elderly Medical Inpatients: Does It Have Utility in the Identification of Delirium? J Geriatr Psychiatry Neurol 18:129–133. https://doi.org/10.1177/0891988705277535

3. Albert MS, Levkoff SE, Reilly C et al (1992) The Delirium Symptom Interview: An Interview for the Detection of Delirium Symptoms in Hospitalized Patients. Top Geriatr 5:14–21. https://doi.org/10.1177/002383099200500103

4. Bergeron N, Dubois M-J, Dumont M et al (2001) Intensive Care Delirium Screening Checklist: evaluation of a new screening tool. Intensive Care Med 27:859–864. https://doi.org/10.1007/s001340100909

5. Bettin KM, Maletta GJ, Dysken MW et al (1998) Measuring delirium severity in older general hospital inpatients without dementia. The Delirium Severity Scale. Am J Geriatr Psychiatry Off J Am Assoc Geriatr Psychiatry 6:296–307

6. Björkelund KB, Larsson S, Gustafson L, Andersson E (2006) The Organic Brain Syndrome (OBS) scale: a systematic review. Int J Geriatr Psychiatry 21:210–222. https://doi.org/10.1002/gps.1449

7. Breitbart W, Rosenfeld B, Roth A et al (1997) The memorial delirium assessment scale. J Pain Symptom Manage 13:128–137. https://doi.org/10.1016/S0885-3924(96)00316-8

8. Cacchione PZ (2002) Four Acute Confusion Assessment Instruments: Reliability and Validity for Use in Long-Term Care Facilities. J Gerontol Nurs 28:12–19. https://doi.org/10.3928/0098-9134-20020101-05

9. Christensen KJ, Bettin KM, Jilk KM et al (1996) Neuropsychological Tests for Monitoring Delirium Severity in Elderly Patients: Am J Geriatr Psychiatry 4:69–76. https://doi.org/10.1097/00019442-199600410-00008

10. Dosa D, Intrator O, McNicoll L et al (2007) Preliminary Derivation of a Nursing Home Confusion Assessment Method Based on Data from the Minimum Data Set: NURSING HOME CONFUSION ASSESSMENT MODEL. J Am Geriatr Soc 55:1099–1105. https://doi.org/10.1111/j.1532-5415.2007.01239.x

11. Emerson G, Carlson R, Nicolson S, Han J (2014) 11 The Clinical Utility of the Clock Drawing Test in Detecting Delirium in Older Emergency Department Patients. Ann Emerg Med 64:S5. https://doi.org/10.1016/j.annemergmed.2014.07.036

12. Eriksson M, Samuelsson E, Gustafson Y et al (2002) Delirium after Coronary Bypass Surgery Evaluated by the Organic Brain Syndrome Protocol. Scand Cardiovasc J 36:250–255. https://doi.org/10.1080/14017430260180436

13. Erkinjuntti T, Sulkava R, Wikström J, Autio L (1987) Short Portable Mental Status Questionnaire as a Screening Test for Dementia and Delirium Among the Elderly. J Am Geriatr Soc 35:412–416. https://doi.org/10.1111/j.1532-5415.1987.tb04662.x

14. Fick DM, Inouye SK, Guess J et al (2015) Preliminary development of an ultrabrief two-item bedside test for delirium. J Hosp Med 10:645–650. https://doi.org/10.1002/jhm.2418

15. Franco JG, Ocampo MV, Velásquez-Tirado JD et al (2020) Validation of the Delirium Diagnostic Tool-Provisional (DDT-Pro) With Medical Inpatients and Comparison With the Confusion Assessment Method Algorithm. J Neuropsychiatry Clin Neurosci 32:213–226. https://doi.org/10.1176/appi.neuropsych.18110255

16. Funk SG (1992) Key aspects of elder care: managing falls, incontinence, and cognitive impairment. Springer Pub. Co, New York

17. Garcia Nuñez D, Boettger S, Meyer R et al (2019) Validation and Psychometric Properties of the German Version of the Delirium Motor Subtype Scale (DMSS). Assessment 26:1573–1581. https://doi.org/10.1177/1073191117744047

18. Gemert van LA, Schuurmans MJ (2007) The Neecham Confusion Scale and the Delirium Observation Screening Scale: Capacity to discriminate and ease of use in clinical practice. BMC Nurs 6:3. https://doi.org/10.1186/1472-6955-6-3

19. Grover S, Mattoo SK, Aarya KR et al (2013) Replication analysis for composition of the Delirium Motor Subtype Scale (DMSS) in a referral cohort from Northern India. Psychiatry Res 206:68–74. https://doi.org/10.1016/j.psychres.2012.08.034

20. Han JH, Vasilevskis EE, Schnelle JF et al (2015) The Diagnostic Performance of the Richmond Agitation Sedation Scale for Detecting Delirium in Older Emergency Department Patients. Acad Emerg Med Off J Soc Acad Emerg Med 22:878–882. https://doi.org/10.1111/acem.12706

21. Han JH, Wilson A, Vasilevskis EE et al (2013) Diagnosing Delirium in Older Emergency Department Patients: Validity and Reliability of the Delirium Triage Screen and the Brief Confusion Assessment Method. Ann Emerg Med 62:457–465. https://doi.org/10.1016/j.annemergmed.2013.05.003

22. Hart RP, Levenson JL, Sessler CN et al (1996) Validation of a Cognitive Test for Delirium in Medical ICU Patients. Psychosomatics 37:533–546. https://doi.org/10.1016/S0033-3182(96)71517-7

23. Hendry K, Quinn TJ, Evans JJ, Stott DJ (2015) Informant single screening questions for delirium and dementia in acute care – a cross-sectional test accuracy pilot study. BMC Geriatr 15:17. https://doi.org/10.1186/s12877-015-0016-1

24. Inouye SK, van Dyck CH, Alessi CA et al (1990) Clarifying confusion: the confusion assessment method. A new method for detection of delirium. Ann Intern Med 113:941–948. https://doi.org/10.7326/0003-4819-113-12-941

25. Inouye SK, Kosar CM, Tommet D et al (2014) The CAM-S: Development and Validation of a New Scoring System for Delirium Severity in 2 Cohorts. Ann Intern Med 160:526. https://doi.org/10.7326/M13-1927

26. Inouye SK, Leo-Summers L, Zhang Y et al (2005) A chart-based method for identification of delirium: validation compared with interviewer ratings using the confusion assessment method. J Am Geriatr Soc 53:312–318. https://doi.org/10.1111/j.1532-5415.2005.53120.x

27. de Jonghe JFM, Kalisvaart KJ, Timmers JFM et al (2005) Delirium-O-Meter: a nurses' rating scale for monitoring delirium severity in geriatric patients. Int J Geriatr Psychiatry 20:1158–1166. https://doi.org/10.1002/gps.1410

28. Katzman R, Brown T, Fuld P et al (1983) Validation of a short Orientation-Memory-Concentration Test of cognitive impairment. Am J Psychiatry 140:734–739. https://doi.org/10.1176/ajp.140.6.734

29. Kean J, Trzepacz PT, Murray LL et al (2010) Initial validation of a brief provisional diagnostic scale for delirium. Brain Inj 24:1222–1230. https://doi.org/10.3109/02699052.2010.498008

30. Lees R, Corbet S, Johnston C et al (2013) Test Accuracy of Short Screening Tests for Diagnosis of Delirium or Cognitive Impairment in an Acute Stroke Unit Setting. Stroke J Cereb Circ. https://doi.org/10.1161/STROKEAHA.113.001724

31. Leonard M, O'Connell H, Williams O et al (2016) Attention, vigilance and visuospatial function in hospitalized elderly medical patients: Relationship to neurocognitive diagnosis. J Psychosom Res 90:84–90. https://doi.org/10.1016/j.jpsychores.2016.09.011

32. Leung JLM, Lee GTH, Lam YH et al (2011) The use of the Digit Span Test in screening for cognitive impairment in acute medical inpatients. Int Psychogeriatr 23:1569–1574. https://doi.org/10.1017/S1041610211000792

33. Lewis LM, Miller DK, Morley JE et al (1995) Unrecognized delirium in ED geriatric patients. Am J Emerg Med 13:142–145. https://doi.org/10.1016/0735-6757(95)90080-2

34. Lin H-S, Eeles E, Pandy S et al (2015) Screening in delirium: A pilot study of two screening tools, the Simple Query for Easy Evaluation of Consciousness and Simple Question in Delirium: A pilot study for screening delirium. Australas J Ageing 34:259–264. https://doi.org/10.1111/ajag.12216

35. Linstedt U, Berkau A, Meyer O et al (2002) Ein kurzer mentaler Test zur Erfassung eines postoperativen Delirs. Ains · Anästhesiol · Intensivmed · Notfallmedizin · Schmerzther 37:205–208. https://doi.org/10.1055/s-2002-25076

36. McCusker J, Cole MG, Dendukuri N, Belzile E (2004) The Delirium Index, a Measure of the Severity of Delirium: New Findings on Reliability, Validity, and Responsiveness: DELIRIUM INDEX. J Am Geriatr Soc 52:1744–1749. https://doi.org/10.1111/j.1532-5415.2004.52471.x

37. Meagher D, Adamis D, Leonard M et al (2014) Development of an abbreviated version of the Delirium Motor Subtyping Scale (DMSS-4). Int Psychogeriatr 26:693–702. https://doi.org/10.1017/S1041610213002585

38. Meagher D, Moran M, Raju B et al (2008) A New Data-Based Motor Subtype Schema for Delirium. J Neuropsychiatry Clin Neurosci 20:185–193. https://doi.org/10.1176/jnp.2008.20.2.185

39. Miller PS, Richardson JS, Jyu CA et al (1988) Association of low serum anticholinergic levels and cognitive impairment in elderly presurgical patients. Am J Psychiatry 145:342–345. https://doi.org/10.1176/ajp.145.3.342

40. Morandi A, McCurley J, Vasilevskis EE et al (2012) Tools to detect delirium superimposed on dementia: a systematic review. J Am Geriatr Soc 60:2005–2013. https://doi.org/10.1111/j.1532-5415.2012.04199.x

41. Morita T, Tsunoda J, Inoue S et al (2001) Communication Capacity Scale and Agitation Distress Scale to measure the severity of delirium in terminally ill cancer patients: a validation study. Palliat Med 15:197–206. https://doi.org/10.1191/026921601678576185

42. Nagley SJ (1986) Predicting and Preventing CONFUSION in Your Patients. J Gerontol Nurs 12:27–31. https://doi.org/10.3928/0098-9134-19860301-09

43. Neelon VJ, Champagne MT, Carlson JR, Funk SG (1996) The NEECHAM Confusion Scale: construction, validation, and clinical testing. Nurs Res 45:324–330. https://doi.org/10.1097/00006199-199611000-00002

44. Ní Chonchubhair A, Valacio R, Kelly J, O'Keefe S (1995) Use of the abbreviated mental test to detect postoperative delirium in elderly people. Br J Anaesth 75:481–482. https://doi.org/10.1093/bja/75.4.481

45. O'keeffe ST (1994) Rating the severity of delirium: The delirium assessment scale. Int J Geriatr Psychiatry 9:551–556. https://doi.org/10.1002/gps.930090708

46. O'Keeffe ST, Gosney MA (1997) Assessing Attentiveness in Older Hospital Patients: Global Assessment Versus Tests of Attention. J Am Geriatr Soc 45:470–473. https://doi.org/10.1111/j.1532-5415.1997.tb05173.x

47. O'Regan NA, Maughan K, Liddy N et al (2017) Five short screening tests in the detection of prevalent delirium: diagnostic accuracy and performance in different neurocognitive subgroups: Screening tests for delirium. Int J Geriatr Psychiatry 32:1440–1449. https://doi.org/10.1002/gps.4633

48. O'Regan NA, Ryan DJ, Boland E et al (2014) Attention! A good bedside test for delirium? J Neurol Neurosurg Psychiatry 85:1122–1131. https://doi.org/10.1136/jnnp-2013-307053

49. Otter H, Martin J, Bäsell K et al (2005) Validity and Reliability of the DDS for Severity of Delirium in the ICU. Neurocrit Care 2:150–158. https://doi.org/10.1385/NCC:2:2:150

50. Rhodius-Meester HFM, van Campen JPCM, Fung W et al (2013) Development and validation of the Informant Assessment of Geriatric Delirium Scale (I-AGeD). Recognition of delirium in geriatric patients. Eur Geriatr Med 4:73–77. https://doi.org/10.1016/j.eurger.2012.11.006

51. Richardson SJ, Davis DHJ, Bellelli G et al (2017) Detecting delirium superimposed on dementia: Diagnostic accuracy of a simple combined arousal and attention testing procedure. Int Psychogeriatr 29:1585–1593. https://doi.org/10.1017/S1041610217000916

52. Robertsson B, Karlsson I, Styrud E, Gottfries CG (1997) Confusional State Evaluation (CSE): An instrument for measuring severity of delirium in the elderly. Br J Psychiatry 170:565–570. https://doi.org/10.1192/bjp.170.6.565

53. Rockwood K, Goodman J, Flynn M, Stolee P (1996) Cross-validation of the Delirium Rating Scale in older patients. J Am Geriatr Soc 44:839–842. https://doi.org/10.1111/j.1532-5415.1996.tb03745.x

54. Rosen J, Sweet RA, Mulsant BH et al (1994) The Delirium Rating Scale in a psychogeriatric inpatient setting. J Neuropsychiatry Clin Neurosci 6:30–35. https://doi.org/10.1176/jnp.6.1.30

55. Rosgen B, Krewulak K, Demiantschuk D et al (2018) Validation of Caregiver-Centered Delirium Detection Tools: A Systematic Review. J Am Geriatr Soc 66:1218–1225. https://doi.org/10.1111/jgs.15362

56. Sala SD, Laiacona M, Spinnler H, Ubezio C (1992) A cancellation test: its reliability in assessing attentional deficits in Alzheimer’s disease. Psychol Med 22:885–901. https://doi.org/10.1017/S0033291700038460

57. Salih SA, Paul S, Klein K et al (2012) Screening for delirium within the interrai acute care assessment system. J Nutr Health Aging 16:695–700. https://doi.org/10.1007/s12603-012-0074-4

58. Shulman RW, Kalra S, Jiang JZ (2016) Validation of the Sour Seven Questionnaire for screening delirium in hospitalized seniors by informal caregivers and untrained nurses. BMC Geriatr 16:44. https://doi.org/10.1186/s12877-016-0217-2

59. Strub RL, Black FW, Black FW (1993) The mental status examination in neurology, 3. ed. Davis, Philadelphia

60. Thomas C, Kreisel SH, Oster P et al (2012) Diagnosing Delirium in Older Hospitalized Adults with Dementia: Adapting the Confusion Assessment Method to International Classification of Diseases, Tenth Revision, Diagnostic Criteria. J Am Geriatr Soc 60:1471–1477. https://doi.org/10.1111/j.1532-5415.2012.04066.x

61. Tieges Z, Stíobhairt A, Scott K et al (2015) Development of a smartphone application for the objective detection of attentional deficits in delirium. Int Psychogeriatr 27:1251–1262. https://doi.org/10.1017/S1041610215000186

62. Tieges Z, Stott DJ, Shaw R et al (2020) A smartphone-based test for the assessment of attention deficits in delirium: A case-control diagnostic test accuracy study in older hospitalised patients. PLOS ONE 15:e0227471. https://doi.org/10.1371/journal.pone.0227471

63. Treloar AJ, Macdonald AJ (1997) Outcome of delirium: Part 1. Outcome of delirium diagnosed by DSM-III-R, ICD-10 and CAMDEX and derivation of the Reversible Cognitive Dysfunction Scale among acute geriatric inpatients. Int J Geriatr Psychiatry 12:609–613. https://doi.org/10.1002/(sici)1099-1166(199706)12:6<609::aid-gps553>3.0.co;2-l

64. Trzepacz PT (1999) The Delirium Rating Scale. Its use in consultation-liaison research. Psychosomatics 40:193–204. https://doi.org/10.1016/S0033-3182(99)71235-1

65. Trzepacz PT, Baker RW, Greenhouse J (1988) A symptom rating scale for delirium. Psychiatry Res 23:89–97. https://doi.org/10.1016/0165-1781(88)90037-6

66. Trzepacz PT, Mittal D, Torres R et al (2001) Validation of the Delirium Rating Scale-Revised-98: Comparison With the Delirium Rating Scale and the Cognitive Test for Delirium. J Neuropsychiatry Clin Neurosci 13:229–242. https://doi.org/10.1176/jnp.13.2.229

67. Vermeersch PE (1990) The clinical assessment of confusion-A. Appl Nurs Res ANR 3:128–133. https://doi.org/10.1016/s0897-1897(05)80132-6

68. Voyer P, Champoux N, Desrosiers J et al (2015) Recognizing acute delirium as part of your routine [RADAR]: a validation study. BMC Nurs 14:19. https://doi.org/10.1186/s12912-015-0070-1

69. Williams MA (1991) Delirium/acute confusional states: evaluation devices in nursing. Int Psychogeriatr 3:301–308. https://doi.org/10.1017/s1041610291000741

70. Yadav K, Boucher V, Carmichael P-H et al (2020) Serial Ottawa 3DY assessments to detect delirium in older emergency department community dwellers. Age Ageing 49:130–134. https://doi.org/10.1093/ageing/afz144

71. Yu A, Teitelbaum J, Scott J et al (2013) Evaluating Pain, Sedation, and Delirium in the Neurologically Critically Ill—Feasibility and Reliability of Standardized Tools: A Multi-Institutional Study. Crit Care Med 41:2002–2007. https://doi.org/10.1097/CCM.0b013e31828e96c0
